# Application of RNA-seq for single nucleotide variation identification in a cohort of patients with hypertrophic cardiomyopathy

**DOI:** 10.1038/s41598-025-03226-x

**Published:** 2025-05-29

**Authors:** Anastasia Chumakova, Ivan Vlasov, Elena Filatova, Anna Klass, Andrey Lysenko, Gennady Salagaev, Maria Shadrina, Petr Slominsky

**Affiliations:** 1 National Research Centre “Kurchatov Institute”, Kurchatov sq. 2, Moscow, 123182 Russia; 2 Petrovsky National Research Center of Surgery, Abrikosovsky Ln 2, Moscow, 119991 Russia

**Keywords:** Genetics, Molecular biology, Genetics research

## Abstract

A variety of techniques for DNA sequencing, such as specific gene sequencing, whole genome sequencing, or exome sequencing, are currently used to detect single nucleotide variations (SNVs). Although RNA-seq can be used to identify SNVs, studies that employ this approach are uncommon, and those that do often rely on outdated mapping methods or methods that are more suitable for genomic and exomic alignment. In this work, our aim is to apply modern RNA-seq specific alignment method in order to identify SNV in a cohort of HCMP patients, and characterize those SNV to gain insight into possible mechanisms of HCMP pathogenesis. The algorithm of identification of SNV based on transcriptomic sequencing data has been developed and evaluated. The algorithm was evaluated and the optimal quality threshold was determined based on allelic discrimination for the rs397516037 mutation (*MYBPC3* c.3697 C > T) among patients. A total of 42,809 SNVs with a quality of 75 or higher were identified in 48 transcriptomes of hypertrophic cardiomyopathy (HCMP) myocardial tissue. Verification of missense and nonsense variants in key HCMP genes using Sanger sequencing confirmed the accuracy of the pipeline results. To identify variants potentially associated with HCMP pathogenesis, a filtration process was conducted based on minor allele frequency, substitution prediction score and ClinVar outcome. 214 missense mutations and 6 nonsense mutations were selected. Together with nonsense mutations, 19 mutations meeting the strictest SIFT and PolypPhen criteria were identified as potential factors influencing HCMP pathogenesis. We have developed and validated a method for identifying SNVs based on transcriptomic data, which can be used to identify putative pathogenic variants. We identified mutations in key HCMP genes *MYBPC3* and *MYH7* in a cohort of patients. We also found potentially pathologic mutations in genes *ANXA6* and *FEM1 A* and obtained data supporting the role of *NEBL* in myocardial diseases. This method would be useful in analyzing transcriptomic data available in the Gene Expression Omnibus, but should be used with caution as we have tested it on a specific disease.

**Supplementary Information:**

The online version contains supplementary material available at 10.1038/s41598-025-03226-x.

## Introduction

Various forms of genomic DNA sequencing are presently used for identification of single nucleotide variations (SNVs) associated with hereditary diseases. These methods vary from sequencing of particular genes in precisely diagnosed and well-studied monogenic diseases up to full genome sequencing for diseases with an unidentified genetic component. A cheaper alternative for full genome sequencing is represented by exome sequencing, clinical exome sequencing, and assays targeting a set of particular single nucleotide polymorphisms (SNPs), associated with various diseases with hereditary components^1–3^.

Full exome sequencing can identify variations in coding regions of a genome or in closely adjacent segments of intron regions, while not investigating intergenic noncoding regions and most of the introns. This omission of most noncoding regions is generally considered well justified, as the majority of currently known mutations and polymorphisms associated with or causative of hereditary diseases are located in coding regions. These mutations typically result in an amino acid substitution (commonly referred to as a ‘missense mutation’), the appearance of a new stop codon (a ‘nonsense mutation’), or shifts in the open reading frame.

The variants in coding regions are likely to have a higher effect size, and their functional consequences are easier to interpret. For common diseases, however, causal variants are often regulatory, affecting the expression of nearby genes. This makes the interpretation of intergenic variants more challenging. While emphasis can be placed on coding variants, the significance of noncoding variants should not be underestimated.

Since those kinds of mutations can only be located in coding regions of mRNA, it is possible to identify them using transcriptome sequencing. Identifying variations in transcripts could allow for identification of both somatic and germline mutations and can simultaneously provide information on transcription levels of mutated mRNA. It is also worth noting that transcribed regions of the genome comprise only a small fraction of the full genome, which allows higher coverage of these regions with smaller number of reads and, consequently, lesser expenses on sequencing^4^. Also, since the RNA for transcriptome sequencing is usually extracted from a particular tissue of interest in the pathogenesis of an investigated disease (e.g., heart tissue in hypertrophic cardiomyopathy (HCMP)), genes that are not expressed in this tissue and, therefore, would likely not be related in any way to disease pathogenesis would be excluded from analysis automatically. Full transcriptome sequencing is widely used in investigations of various diseases. The results of many such investigations are publicly available in open databases, such as Gene Expression Omnibus^5^. These accumulated data could be used for identification of novel mutations and disease-associated variants, which would expand the current understanding of genetic components of a wide range of diseases. Despite using RNA-seq in order to identify SNV being a possibility, studies employing this approach are quite rare, and ones that do often use deprecated methods of mapping^6,7^ or methods of mapping which are better suited for genomic and exomic alignment^4^. Therefore, in this work, our aim is to apply modern RNA-seq specific alignment method in order to identify SNV in a cohort of HCMP patients, and characterize those SNV to gain insight into possible mechanisms of HCMP pathogenesis.

HCMP is a disease characterized by hypertrophy of the left ventricle and subsequent clinical consequences, including sudden cardiac death (SDC), heart failure, and atrial fibrillation, followed by embolic stroke^8–10^. HCMP is usually considered to be a monogenic disease with a heterogenic hereditary component and autosomal dominant type of inheritance^10^. Most cases of HCMP are associated with pathogenic variants in the main sarcomeric genes (*MYH7*, *MYBPC3*, *TNNT2*, *TNNI3*, *MYL2*, *MYL3*, *TPM1*, *ACTC1*)^9,10^. However, these are not the only genes associated with HCMP pathogenesis, and currently over 1400 different associated mutations in dozens of additional genes have been identified (Chakova et al., 2017). The genetic heterogeneity of HCMP makes it a fitting subject of inquiry in investigation of RNA-seq’s potential in identification of novel, pathogenically significant SNVs. Our research group is currently working on transcriptome profiling of the myocardium of HCMP patients. The goals of the current study are to develop a pipeline for SNV identification from RNA-seq data and apply this pipeline to identification of putative pathologically significant SNV using accumulated RNA-seq data.

## Materials and methods

### Ethical compliance

The study was conducted in accordance with the World Medical Assembly Declaration of Helsinki. The study was approved by the Ethics Committees of Institute of Molecular Genetics of National Research Centre “Kurchatov Institute” (Protocol №22/5, 16.12.2022). Written informed consent was obtained from all participating patients and families.

### Patient cohort

Forty-eight unrelated adult patients with HCMP have been used in this study. The average age of a patient was 52 ± 24.48 years. The ratio of male to female patients was 1: 1 (m/f). Characteristics of the patients enrolled in the study are presented in the Supplementary Table 2. Patients were diagnosed in concordance with the *ESC Guidelines* on diagnosis and management of hypertrophic cardiomyopathy (a ≥ 15-mm-thick interventricular septum with no other identified causes of hypertrophy)^11^. Sixteen out of 48 patients had an older relative with SCD (6) and/or HCMP (11). Forty-three out of 48 patients have been tested for rs397516037 mutation (*MYBPC3* c.3697 C > T), using TaqMan allelic discrimination real-time PCR assay for detection of single nucleotide substitution. Primer and probe sequences used in the assay are found in Supplementary Table 1 on page 3 on the Supplementary Material.

### Myocardium tissue preparation, RNA extraction, and RNA sequencing

Myocardium bioptates were placed in RNALater (Invitrogen, United States) solution immediately after extraction, then stored at + 4 °C for 24 h, and then refrigerated at −20 °C for transportation. Subsequently, samples were stored at −80 °C. Total RNA was extracted using TRIzol (Invitrogen, United States), in accordance with manufacturer’s recommendations. The quality and quantity of total RNA were measured using BioAnalyser with RNA 6000 Nano Kit (Agilent, United States). A poly(A) fraction of RNA was extracted, and libraries for sequencing were prepared using an NEBNext^®^ mRNA Library Prep Reagent Set (NEB, United States). Sequencing was performed by Genoanalitica (Russia, Moscow) using an HiSeq 1500 (Illumina, United States), generating no less than 15 million 50 bp reads.

### RNA-seq data preparation and alignment

Removal of ambiguous and low-quality nucleotides from FASTQ was performed using an AdapterRemovalV2^12^. Read alignment to the GRCH38 genome was conducted using the “rsem-calculate-expression” command in RSEM^13^ and STAR^14^ tools with an enabled “-star” option.

### SNV analysis

BAM files obtained in the previous step were sorted using «samtools sort» from SAMtools^15^. Sorted BAM files were converted into pileup with the «bcftools mpileup» command from BCFtools^16^. An SNV call was performed using the «bcftools call» command with subsequent filtration with the «bcftools filter -i QUAL» command. Obtained VCF files were compressed and indexed using “bgzip” and “tabix” from the HTSlib library^17^. Then variants were sorted by quality. A quality threshold of 75 was selected based on concordance of call and filtration results to previously obtained TaqMan allelic discrimination results on rs397516037 in such a way that neither false positives or false negatives were identified in the genomic position of rs397516037.

### Genomic DNA extraction

Genomic DNA extraction from peripheral blood and myocardium tissues (in cases in which no blood was available) was performed using a Quick-DNA™ Miniprep Plus Kit (Zymo Research Corp., United States) in accordance with the manufacturer’s recommendations. DNA concentration was determined using a Qubit 3.0 0 fluorometer and Qubit dsDNA BR (Broad-Range) Assay Kit (Invitrogen™, United States).

### SNV confirmation using Sanger sequencing

SNV was confirmed in eight patients, identified using RNA-seq, using Sanger sequencing. Primers were designed based on GRCh38 genome assembly using Premier Biosoft International Beacon Designer 7.0 (Palo Alto, United States) (Supplementary Table 1 on page 3 on the Supplementary Material). Target sequence amplification was performed using the QuantStudio 3 (Thermo Fisher Scientific, United States) and PCR reagents (Sintol and DNK-Sintez (Russia), Thermo Fisher Scientific (United States)). The reaction mix included: 3 µl of buffer solution (x10), 3 µl dNTP (2 nmol/µl each), 3 µl MgCl_2_ (25 nmol/µl), 1 µl of each primer (10 pmol/µl), 1 µl of each probe (5 pmol/µl), 0.2 µl Taq-polymerase (5 U/µl), and up to 30 µl of ultrapure water. The amplification cycle protocol was as following: 180 s at 95 °C, then 40 cycles of 5 s at 95 °C and 20 s at 60 °C. Obtained target fragments were separated using gel electrophoresis in 2% agarose gel, cut, and purified with a Cleanup S-Cap kit (Evrogen, Russia). Sanger sequencing was performed by Evrogen (Russia, Moscow). Sequencing was performed in both the forward and backward directions where possible.

## Results

**Fig. 1:**
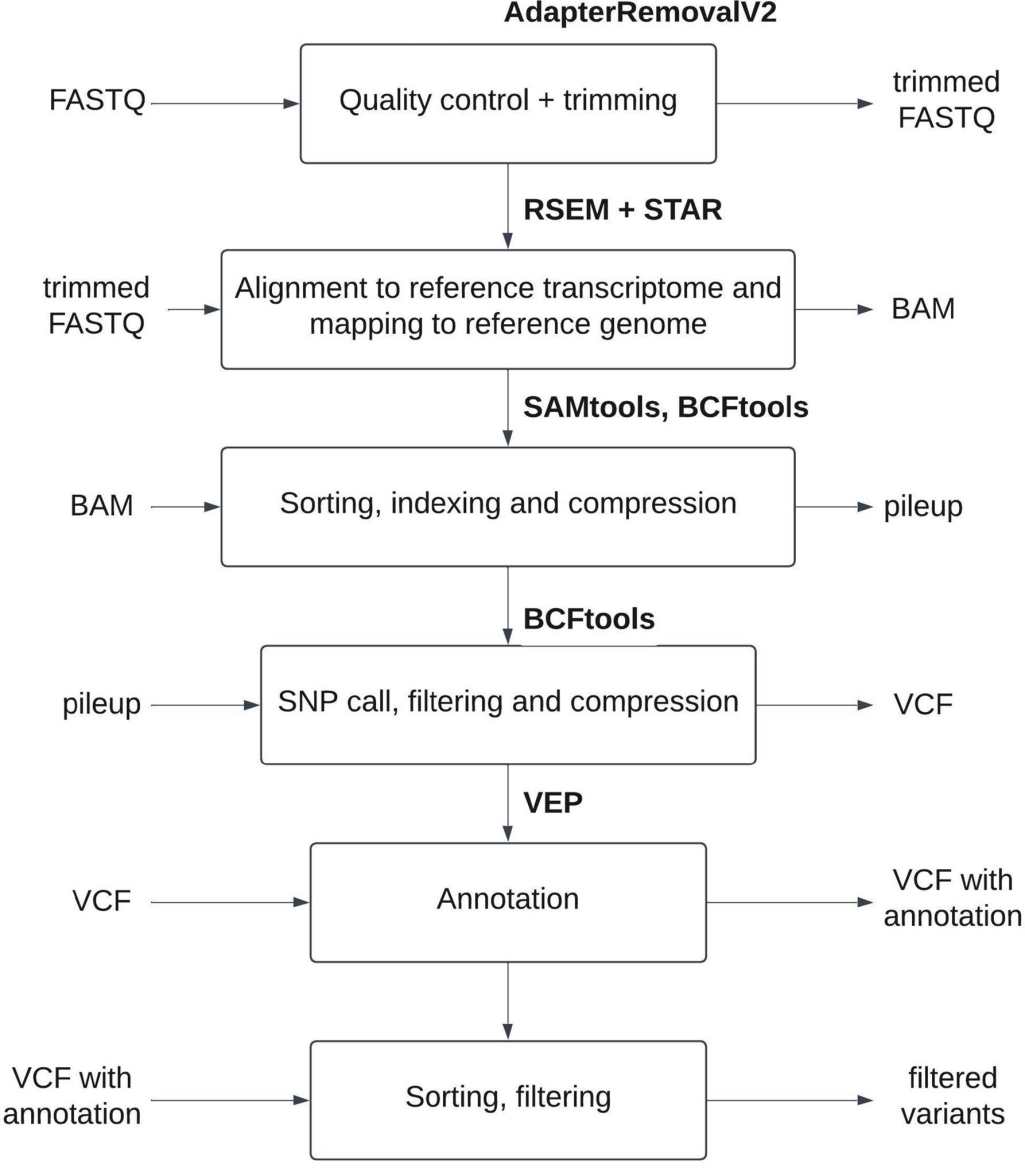
Pipeline for SNV identification from RNA-seq.

The pipeline developed for SNV identification from RNA-seq is presented in Fig. 1.

AdapterRemovalV2 is used for removal of ambiguous and low-quality nucleotides from the fastq libraries. Read mapping to the reference genome is performed using STAR and RSEM by the «rsem-calculate-expression» command with the «-star» option enabled. Alignment rates for each alignment obtained are presented in Supplementary Table 3 on page 4 on the Supplementary Material. Bam files are then sorted using the «samtools sort» command for SAMtools. Sorted bam files are then converted into pileup format using «bcftools mpileup», and then SNVs are called from pileup into VCF format using the «bcftools call» command for BCFtools. VCF files are filtered based on quality with the «bcftools filter -i QUAL» command, and then compressed and indexed using bgzip and tabix from the HTSlib library^17^.

Called variants were split into groups based on quality (< 20, 20–50, 50–75, 75–100, 100–120, 120–140, > 140) in order to select a proper threshold for quality filtration.

In order to evaluate the developed algorithm and aid in threshold selection, allelic discrimination data for rs397516037 (*MYBPC3* c.3697 C > T) mutation in patient cohort was obtained. Four patients were identified as carriers of heterozygous rs397516037 (C/T). Thirty-nine patients had a major allele in a homozygous (CC) state. Five patients were not tested due to an absence of blood samples. Using these data, the upper threshold of quality for filtration has been chosen, based on the minimal quality at which false negatives were observed, of 120 quality. Notably, no false positives were observed across all quality thresholds applied.

For each quality group, a number of randomly selected variants were manually checked using IGV. The minimal threshold of quality was selected in such a way that at least five reads were found for each of the alleles in heterozygous states. Based on this, the minimal threshold of quality for filtration was determined to be 75. Since no false positives were detected in the previously verified rs397516037 genomic position at the threshold of 75, this threshold was used for further analysis.

A total of 42 809 SNVs with 75 or higher quality were identified in 48 transcriptomes of HCMP myocardial. Next, in order to identify variants, potentially associated with HCMP pathogenesis, a filtration based on minor allele frequency (MAF), substitution prediction score and ClinVar outcome have been conducted. 35,574 SNV were filtered out based on having higher than 1% MAF in either 1000 genomes, Gnomad genome or Gnomad exome databases. Then, out of remaining SNV missense (214) (Supplementary Table 4) and nonsense mutations (6) (Table 1) have been selected.

Two groups of missense mutations were filtered based on SIFT and PolyPhen scores – a group (19) with maximum PolypPhen (1) and SIFT (0) score (Table 2), and a wider selection of missenses (214) with PolypPhen predictions being “possibly_damaging” or “probably_damaging” and SIFT predictions being either “deleterious” or “deleterious_low_confidence” (Supplementary Table 4). Together with nonsense mutations, 19 mutations filtered by the strictest SIFT and PolypPhen criteria can be considered as potential factors, impacting pathogenesis of HCMP (Table 2).

**Table 1:** Identified nonsense mutations.

Gene	Location of variant	Ensembl Transcript ID	Replacement of AA	rs (dbSNP (NCBI))	MAF (gnomADe_NFE_AF)	*N*
*NEBL*	10_20888199_G/C	ENST00000377122	p.Y89*	rs147622517	0.002341	1
*MYBPC3*	11_47332189_G/A	ENST00000256993, ENST00000399249, ENST00000545968	p.Q1233*	rs397516037	0.0000177	4
*MYBPC3*	11_47341180_C/A	ENST00000256993, ENST00000399249, ENST00000544791, ENST00000545968	p.E619*	NA	NA	1
*FEM1 A*	19_4793280_G/T	ENST00000269856	p.E476*	rs371110900	0.00001782	1
*DNAAF3*	19_55165815_C/A	ENST00000528412, ENST00000534214	p.E91*	NA	NA	1
*VDAC3*	8_42396675_C/G	ENST00000518495	p.S46*	NA	NA	11

AA – amino acid; MAF - minor allele frequency; N - number of patients in cohort with this mutation; NA - not available.

**Table 2:** Identified missense mutations with polypphen score of 1 and SIFT score of 0.

Gene	Location of variant	Ensembl Transcript ID	Replacement of AA	rs (dbSNP (NCBI))	MAF (gnomADe_NFE_AF)	*N*
*MRPL37*	1_54216226_T/C	ENST00000336230, ENST00000360840, ENST00000398219, ENST00000605337	p.L228/359/144P	rs150955584	0.0001231	1
*PRDX6*	1_173486337_T/C	ENST00000340385	p.L161P	NA	NA	1
*PRDX3*	10_119172416_C/A	ENST00000298510	p.G173 C	rs11554923	0.007621	1
*TM7SF2*	11_65115034_C/T	ENST00000279263, ENST00000345348, ENST00000525385	p.P282/253L	rs187240326	0.001878	1
*CRYAB*	11_111911694_G/C	ENST00000533971	p.R11G	rs781902168	NA	1
*BCO2*	11_112202066_A/G	ENST00000357685, ENST00000438022, ENST00000526088, ENST00000531169, ENST00000532593	p.H357/323/252R	rs143616587	0.005767	1
*PLBD1*	12_14506992_C/T	ENST00000240617	p.R438Q	rs75778757	0.007259	2
*MYH7*	14_23415652_G/A	ENST00000355349	p.R1712 W	rs121913650	0	1
*MYH7*	14_23429005_G/A	ENST00000355349	p.R453 C	rs121913625	NA	1
*FAM234 A*	16_265002_C/T	ENST00000301678, ENST00000399932	p.R547 W	rs201598421	0.003405	1
*BCKDHA*	19_41424582_T/A	ENST00000269980	p.Y438 N	rs137852870	0.000143	1
*ZNF880*	19_52384818_G/T	ENST00000422689	p.C413 F	rs12975097	0.00412	2
*ACSS1*	20_25007898_C/T	ENST00000323482, ENST00000537502	p.R645/524Q	rs377542546	0.00006156	1
*RPN1*	3_128632079_G/A	ENST00000296255, ENST00000497289	p.H238/66Y	rs138936459	0.0006242	1
*ANXA6*	5_151133117_C/T	ENST00000354546, ENST00000523714, ENST00000700367	p.R206/174H	rs759582371	0	1
*HEBP2*	6_138405194_C/T	ENST00000367697	p.T51M	rs775665563	0.00004396	1
*DNAJC30*	7_73683272_T/C	ENST00000395176	p.Y51 C	rs61732167	0.001213	1
*ASAH1*	8_18071334_C/T	ENST00000636171, ENST00000636455, ENST00000636537, ENST00000637638	p.R61/77 K	NA	NA	7
*MT-ND1*	MT_3460_G/A	ENST00000361390	p.A52 T	rs199476118	NA	1

AA – amino acid; MAF - minor allele frequency; N - number of patients in cohort with this mutation; NA - not available.

In order to further check the results obtained using the pipeline, missense and nonsense variants in hallmark HCMP genes *MYBPC3* and *MYH7* were selected (Table 3). Of the 8 obtained, 7 were verified using Sanger sequencing, with the exception of one missense mutation in *MYH7* (p.D1378G) for which there was not enough material for verification. The results of this verification can be found in Supplementary Fig. 1 on page 1 on the Supplementary Material.

**Table 3:** Missense and nonsense mutations in *MYBPC3* and *MYH7* genes identified in our patient cohort using the developed pipeline.

Gene	Location of variant	Ensembl Transcript ID	Replacement of AA	rs (dbSNP (NCBI))	MAF (gnomADe_NFE_AF)	Result	*N*
***MYBPC3***	11_47341180_C/A	ENST00000256993, ENST00000399249, ENST00000544791, ENST00000545968	p.Е619*	NA	NA	stop_gained, NMD_transcript_variant	1
***MYH7***	14_23415221_T/A	ENST00000355349	p.H1778L	NA	NA	missense_variant	1
	14_23415652_G/A	ENST00000355349	p.R1712 W	rs121913650	0	missense_variant	1
	14_23418246_T/C	ENST00000355349	p.D1378G	NA	NA	missense_variant	1
	14_23422309_T/C	ENST00000355349	p.E1039G	rs199573700	0.00003517	missense_variant	1
	14_23424876_G/A	ENST00000355349	p.R858 C	rs2754158	0.00001759	missense_variant	1
	14_23426834_G/A	ENST00000355349	p.R663 C	rs397516127	0	missense_variant	1
	14_23429005_G/A	ENST00000355349	p.R453 C	rs121913625	NA	missense_variant	1

AA – amino acid; MAF - minor allele frequency; N - number of patients in cohort with this mutation; NA - not available.

## Discussion

At present, very few studies considering use of RNA-seq for SNV identification are published. In a study by Chepelev et al., the high cost of full genome sequencing, as compared to transcriptome sequencing, is emphasized^4^. It is worth noting that exome sequencing is also cheaper than full genome sequencing. However, this is most likely not considered in the study by Chepelev because of timing, since the paper by Chepelev et al. was published only a month after a study that introduced exome sequencing as a cheaper alternative for full genome sequencing for identification of novel variants^18^, and both papers were likely in progress simultaneously. However, Chepelev et al. also considers the ability to simultaneously evaluate both presence of SNVs and changes in expression as advantages of transcriptome sequencing as compared to full genome sequencing^4^, which would also be an advantage of transcriptome sequencing as compared to exome sequencing.

In the study by Chepelev et al. (Chepelev et al., 2009), 20 SNVs were selected to be verified. Regions containing 18 out of 20 of these SNVs were successfully amplified and sequenced using Sanger sequencing, with 16 out of 18 being confirmed^4^. One of the unconfirmed SNVs was actually confirmed to be present in mRNA, based on cDNA sequencing. This result demonstrates that changes in the structure of the protein can emerge as a result of RNA editing^4^. The possibility to identify such variation, which ultimately leads to a change in protein structure without appearing in genomic DNA, can also be considered an advantage of transcriptome sequencing.

In a paper by Cirulli et al.^1^, a comparison between identification of SNVs using transcriptomic sequencing and genomic sequencing on samples derived from the same individuals was conducted. This comparison allowed both evaluating the sensitivity and specificity of transcriptome sequencing for SNV identification and estimating the effects of SNVs on gene expression. When considering all the coding SNVs, RNA-seq has only captured 41% of SNVs captured by genomic sequencing. However, when considering only the genes expressed in the target tissue, the intersection comprised 81% SNV^1^. Overall, 48 740 SNVs were identified in genomic DNA and 40 605 SNVs were identified in cDNA. A total of 19 054 of them were common to both genomic and cDNA^1^. Both sensitivity (number of true positives divided by the sum of true positives and false negatives) and specificity (number of true positives divided by the sum of true positives and false positives) were evaluated for transcriptome sequencing, on the basis of considering SNVs to be correctly identified by genomic DNA sequencing^1^. Quality filter thresholds chosen in the study were optimized in such a way as to maximize both sensitivity and specificity. When considering all the genes, including ones that are not expressed in the target tissue, sensitivity amounted to 0.39 and specificity amounted to 0.47. Identified SNVs were also compared to dbSNV records. It has been noted that 94% of true positives were found in dbSNV, whereas only 23% of false positives and 89% of false negatives were found in dbSNV^1^. Also, the percentage of SNV intersection with dbSNV was found to be inversely correlated with coverage in transcriptomic sequencing a lower proportion of false positive SNVs were found in regions with higher coverage^1^.

We have also analyzed pipelines used in studies, investigating identification of SNV in RNA-seq data, and compared those with our own approach, paying special attention to tools used to map the reads and tools used to call SNV. We have identified 6 appropriate work to compare our own pipeline with – Chepelev et al. 2009, Cirulli et al. 2010, Piskol et al. 2013, Quinn et al. 2013, Liu 2019 and Dou et al. 2024^4,6,7,19–21^. Chepelev et al. 2009^4^ use ELAND by Illumina as a read mapping tool. It is worth noting that ELAND is a tool for genomic alignments. Similarly, Piskol et al. 2013^19^ use BWA for read alignment, which is also a tool developed for genomic/exomic alignment. However, currently a lot of tools have been developed specifically for transcriptome alignment, and are generally considered to work better for that goal, such as Rsubread^22^ and STAR (that is used in our pipeline)^14^, and we believe that using such specialized tools is a better approach. Quinn et al. 2013 and Cirulli et al. 2010 both use Tophat2, which is currently considered deprecated by its developers^23^. Dou et al. 2024^21^ don’t describe the procedure of obtaining mappings in their paper. Finally, Liu et al.^20^ consider both STAR and GSNAP, ultimately ending up using STAR, since based on their data using it leads to higher true positive rate in SNV detection.

For SNV calling, both Chepelev et al. and Dou et al. used the tools that they developed, being Point Mutation Analyzer and Monopogen, respectively. Piskol et al. also use their own filtering algorithm SNiPR, in combination with GATK^24^. Quinn et al. use both Samtools and GATK, while Cirulli et al. only use Samtools. Finally, Liu et al.^20^ compares several different SNV calling methods, including both Samtools, GATK and several others. Their analysis concludes that Samtools is the recommended method of SNV calling. However, it is worth noting that Liu et al. are testing both SNV calling and mapping as applied to scRNA-seq data, and their results might be not perfectly applicable to our research. While we aсknowledge that GATK is widely considered to be the gold standard for SNV-calling, based on Liu et al. comparison and its performance in our own test, we consider that combination of STAR and Samtools is a good choice for SNV calling from RNA-seq data.

In order to check the results obtained using our pipeline, we reviewed and validated a total of 8 SNV using two different methods: the rs397516037 mutation was tested using TaqMan real-time PCR assay with allelic discrimination in 43 patients. 7 missense and nonsense variants of the characteristic HCMP genes *MYBPC3* and *MYH7* verified and confirmed in 8 patients using Sanger sequencing. We have not identified any discrepancies between results of real-time PCR-based allelic determination/Sanger sequencing and RNA-seq data. Therefore, in the limited scope of experimental verification we have conducted, both specificity and sensitivity amount to 1. However, it is worth noting that, due to the small number of verified mutations, this result probably requires further verification. However, it is worth noting that verification of individual mutations with Sanger sequencing or allelic determination is more reliable than verification of them based on genomic sequencing.

Most HCMP-associated genes carry both familial and de novo pathogenic variants^9^. Most of these variants are missense mutations and have dominant hereditary properties^9,25^. Some pathogenic variants are also characterized by incomplete penetrance, which could depend on environmental or/and other genetic factors. A multitude of rare pathogenic variants with average or low penetrance are found in patients with sporadic HCMP and small families with familial HCMP^9,10^. It has been established that most HCMP-related genes code sarcomere or sarcomere-associated proteins. From 70 to 80% of familial cases of HCMP carry mutations in the heavy myosin chain gene *MYH7* and myosin binding protein C gene *MYBPC3*^9,26^. Patients with pathogenic variants in *MYH7* have an increased risk of developing atrial fibrillation, earlier onset of disease, and more severe form of disease overall than patients with pathogenic variants in *MYBPC3*. Also, it has been established that HCMP patients with mutations in *TNNI3* had shorter life expectancy compared to carriers of mutations in either *MYBPC3* or *MYH7*, while HCMP patients with mutation in *TNNC1* had an increased risk of developing a fatal atrial arrhythmia^27^. Based on the prevalence of *MYBPC3* or *MYH7* mutations in cases of HCMP, we have decided to use mutations in those two genes for further verification.

In order to identify SNV, potentially associated with HCMP pathogenesis, we have employed several filters, such as MAF, outcome of mutation, amino acid substitution prediction score (in case of missenses). These filtered lists were then used to evaluate putative mutations based on genes they are situated in and their relevance to HCMP.

Gene *MYBPC3*, encoding myosin binding protein C is considered to be one of the key sarcomere genes, causatively linked to HCMP development^28^. Based on various estimates, 40 to 50% o all HCMP associated mutations are located within this gene^29^, and most of those lead to production of truncated transcripts^30^, which is also the case with nonsense mutations we have identified in *MYBPC3* in our cohort of HCMP patients. Due to cMyBP-C playing a key role in regulation of myosin-actin cross-bridge kinetics, nonsense mutations in 11_47332189_G/A (p.Q1233X) and 11_47341180_C/A (p.E619X)) could lead to haploinsufficiency, which could in turn lead to disturbances in contraction/relaxation cardiomyocytes. It is worth noting that despite not being annotated in dbsnp, 11_47341180_C/A was previously identified in young Russian patents with HCMP^31^, suggesting it might be endemic to this region.

*NEBL* encodes mechanosensitive protein of Z-disc Nebulete^32^, which plays a key role in organization and functioning of myofibrilla, which could imply that early termination of NEBL translation could play a role in HCMP pathogenesis. *NEBL* isn’t considered to be a hallmark HCMP gene, however variants in this gene were previously associated with several types of cardiomyopathies, including HCMP, dilated cardiomyopathy and left ventricular non-compaction cardiomyopathy^33,34^. It has also been shown that mutatuion in NEBL could be a causative in pathogenesis of Brugada syndrome^35^. Based on that, rs147622517 can be either a reason, or, more likely, considering relatively high frequency of this variant in population (0,15%), a risk factor of HCMP pathogenesis.

Nonesense variant rs371110900 which we have identified in our patient cohort is located in *FEM1 A.* Gene *FEM1 A* encodes a part of CRL2 complex FEM1 A which serves as an adaptor for protein ubuqitination by E3 ligase^36^. Based on ClinVar data, rs371110900 has no known associations with cardiovascular diseases, however, it is known that this variant is associated with polycystic ovary syndrome^37^. It is also worth noting that increased expression of *Fem1a* was identified in ischemia-reperfusion in mice^37^, while RNA-seq of human myocardial tissues has shown repression of *FEM1 A* expression after ischemia^38^. Taken together with identification of rs371110900 in HCMP patient, this data suggests that role of *FEM1 A* in normal and pathological heart physiology should be investigated.

Missense mutations, filtered by strictest Polyphen (1) and SIFT (0) scores (Table 3) were also investigated for potential connections to HCMP in literature. Among the genes they are located in, *ANXA6* appears to be the most relevant in this regard. In it, we have identified rs759582371, situated in exone 8 and leading to R206/174H, with undetermined clinical significance in ClinVar. Very high Polyphen and SIFT scores of this mutation are most likely explained by the fact that this is a substituition of aliphatic amino acid to aromatic one with weak basic properties.

ANXA6 protein is the main myocardial annexin from the family of calcium and phospholipid binding membrane proteins, which plays an important role in regulation of endocytosis and exocytosis and supporting the homeostasis of calcium in cardiomyocytes. Using transgenic mice, it has been shown that knockout of ANXA6 leads to dilatational changes in myocardium, whereas it’s increased expression leads to hypertrophic changes^39^. These effects could be linked to its interaction with atrial natriuretic peptide pathways, which participate in vitro in regulation of processes of hypertrophy and apoptosis of H9c2 rat myoblasts^40,41^. ANXA also participates in cholesterol metabolism through interactions with phospholipase A2 and blocking of EGFR-Ras pathway^42^, which could in turn affect myocardiocyte growth. It has also been shown that annexin 6 directly interactis with sarcomeres alpha-actinin, and this interaction affects excitability and contractability of cardiomyocyte^43^. Overall, *ANXA6* plays an important role in cardiac function, which in turn means that mutation in *ANXA6* could potentially be related to HCMP. However, additional research is required in order to establish what causative role it plays, if any.

It is also worth noticing that the same patient that carries rs759582371 also carries a missense mutation in *MYH7*, one of the hallmarks HCMP genes (rs2754158). rs2754158 is also considered to have an established pathogenic effect in HCMP^44–48^.

We can conclude that using RNA-seq for SNV identification, we have successfully identified a number of mutations in HCMP patient cohort, with some even having an established relation to HCMP pathogenesis. Use of transcriptomic sequencing for SNV identification would allow an additional investigation to be conducted of accumulated RNA-seq data on various diseases. It would allow additional data to be procured from already-existing datasets, which have already served their purpose in investigation of transcriptional profiles, as well as allowing the effects of SNVs on gene expression within each sample to be observed. Also, the fact that reads are only assigned to genes expressed in target tissue allows for more efficient use of coverage and, consequently, lesser expenses for sequencing.

However, it is also worth considering the limitations of such an approach. Difficulties with reaching high coverages for transcripts with lower expression levels would probably lead to increased chance of false negative results for SNVs in such genes. An average coverage of no less than 70X for a full exome and no less than 30X for a full genome are usually considered to be standard^49^. In the case of RNA-seq, since the coverage is directly linked to gene expression, reaching such coverages for less-expressed genes appears almost impossible. As such, the aforementioned increased risk of false negatives seems inevitable. Verifying such false negatives is also a complicated task, since it is much easier and cheaper to confirm that an SNV indeed exists than to investigate genetic loci that could have SNVs that have been missed.

Another possible issue with use of RNA-seq for SNV identification in nonsense-mediated decay (NMD). Transcripts carrying NMD mutations can be absent or less abundant in transcriptome due to this very decay^50^, whereas detecting NMD mutations in the case of exome and genome sequencing is not more complicated than any other SNV. In a single case, among the SNVs chosen for verification, in our studies with SNVs with a predicted NMD effect (11_47341180_C/A in Table 1), we have found no observable effect of NMD on transcript abundance (Supplementary Fig. 2). However, this outcome is far from conclusive, and it is still worth considering the possibility of having a false negative result due to NMD in other cases.

It is also worth noting that genes *MYH7* and *MYBPC3* present the best-case scenario for use of RNA-seq for SNV identification, since both genes are very highly expressed in the myocardium. Therefore, the results may not be as good for other diseases and target tissues. This method overall appears to be very well suited for the study of diseases with autosomal-dominant inheritance, linked to mutations that affect the protein structure. Its applicability to the study of diseases with recessive inheritance with other types of causative mutations should be evaluated separately.

## Conclusions

In this study, the method of identification of SNVs based on transcriptome sequencing data has been developed, applied, and verified. Based on our own data and results obtained in previous studies, this method could be used for identification of putative pathogenic variants. We have identified a number of mutations in a cohort of HCMP patients, including ones in the key HCMP genes - *MYBPC3* и *MYH7*. We have also identified potentially pathologic mutations in genes with no previously established relation to *HCMP – ANXA6* and *FEM1 A.* We have also obtained data that supports the possible role of *NEBL* in various myocardial diseases.

The use of such a method would be especially interesting in the context of the vast amounts of already-accumulated transcriptomic data that have yet to be examined in such a way, such as the datasets available, for example, in the Gene Expression Omnibus.

However, the limitations and applicability of this method should also be carefully considered. We have examined it on the basis of disease and type of inheritance, which appears to be very well suited for the method, but, in cases of other diseases and types of inheritance, its applicability should be evaluated on a case-by-case basis.

## Electronic supplementary material

Below is the link to the electronic supplementary material.

Supplementary Material 1

Supplementary Material 2

Supplementary Material 3

## Data Availability

Raw FAST files and processed read count are available from Gene Expression Omnibus (GEO), accession number GSE273325.
