# Supplementary material for: Application of RNA-seq for single nucleotide variation identification in a cohort of patients with hypertrophic cardiomyopathy
Source: Sci Rep. 2025 May 29;15:18788. doi: 10.1038/s41598-025-03226-x (PMC12122699; doi:10.1038/s41598-025-03226-x)
Supplement: Supplementary file 1 — Supplementary Material 1 [file 41598_2025_3226_MOESM1_ESM.pdf]

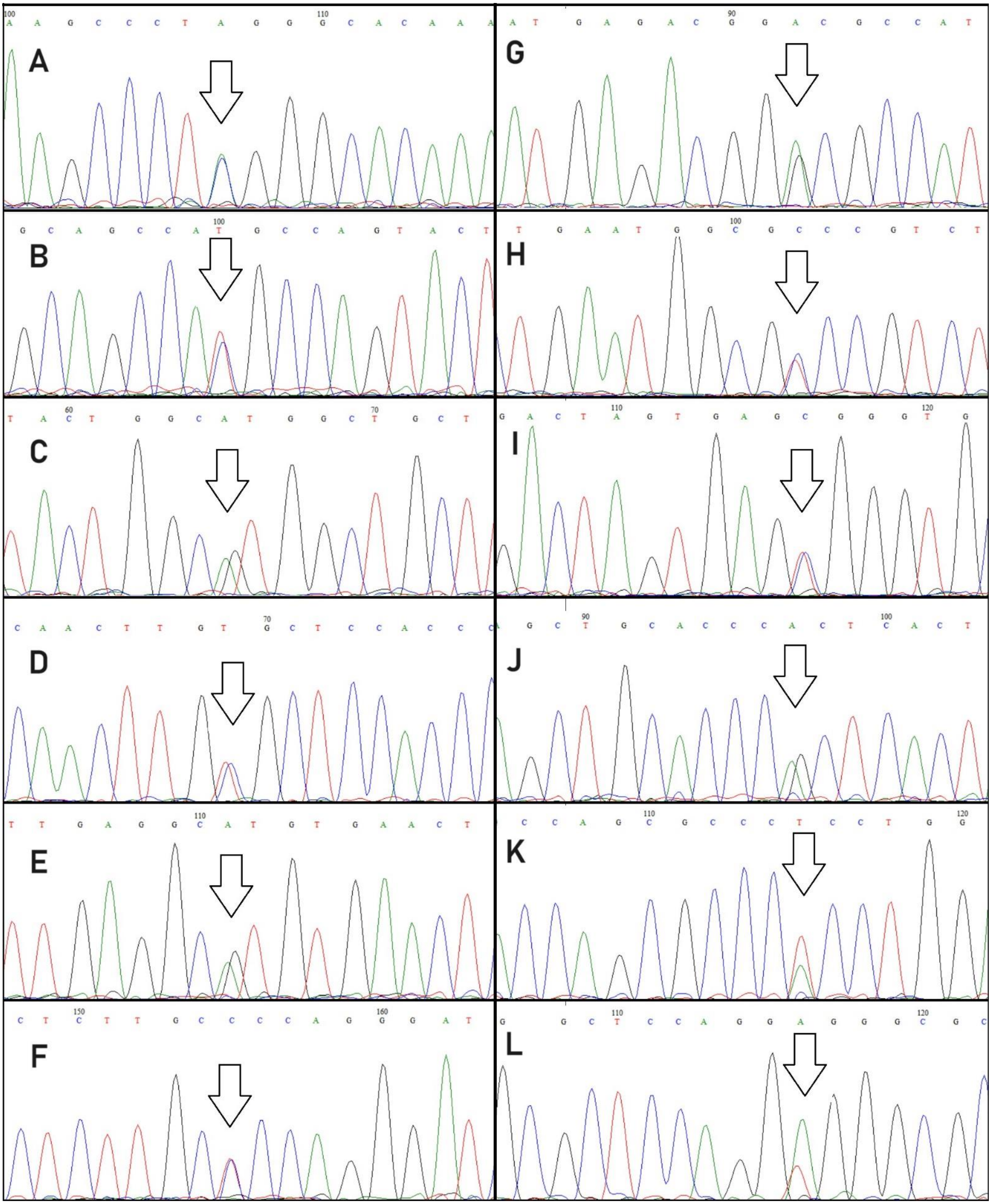

Supplementary figure 1. Sanger sequencing of target regions containing identified SNVs in *MYBPC3* and *MYH7* genes of patients in our patient cohort

A - c.1855G>T (*MYBPC3* gene; read from reverse primer); **B-C** - c.1357C>T (*MYH7* gene; reading from both forward and reverse primer); **D** - c.1987C>T (*MYH7* gene; reading from the forward primer); **E** - c.2572C>T (*MYH7* gene; reading from the reverse primer); **F -** c.3116A>G (*MYH7* gene; reading from the reverse primer); **G-H -** c.4133A>G (*MYH7* gene; reading from both forward and reverse primer); **I-J** - c.5134C>T (*MYH7* gene; reading from both forward and reverse primer); **K-L** - c.5333A>T (*MYH7* gene; reading from both forward and reverse primer).

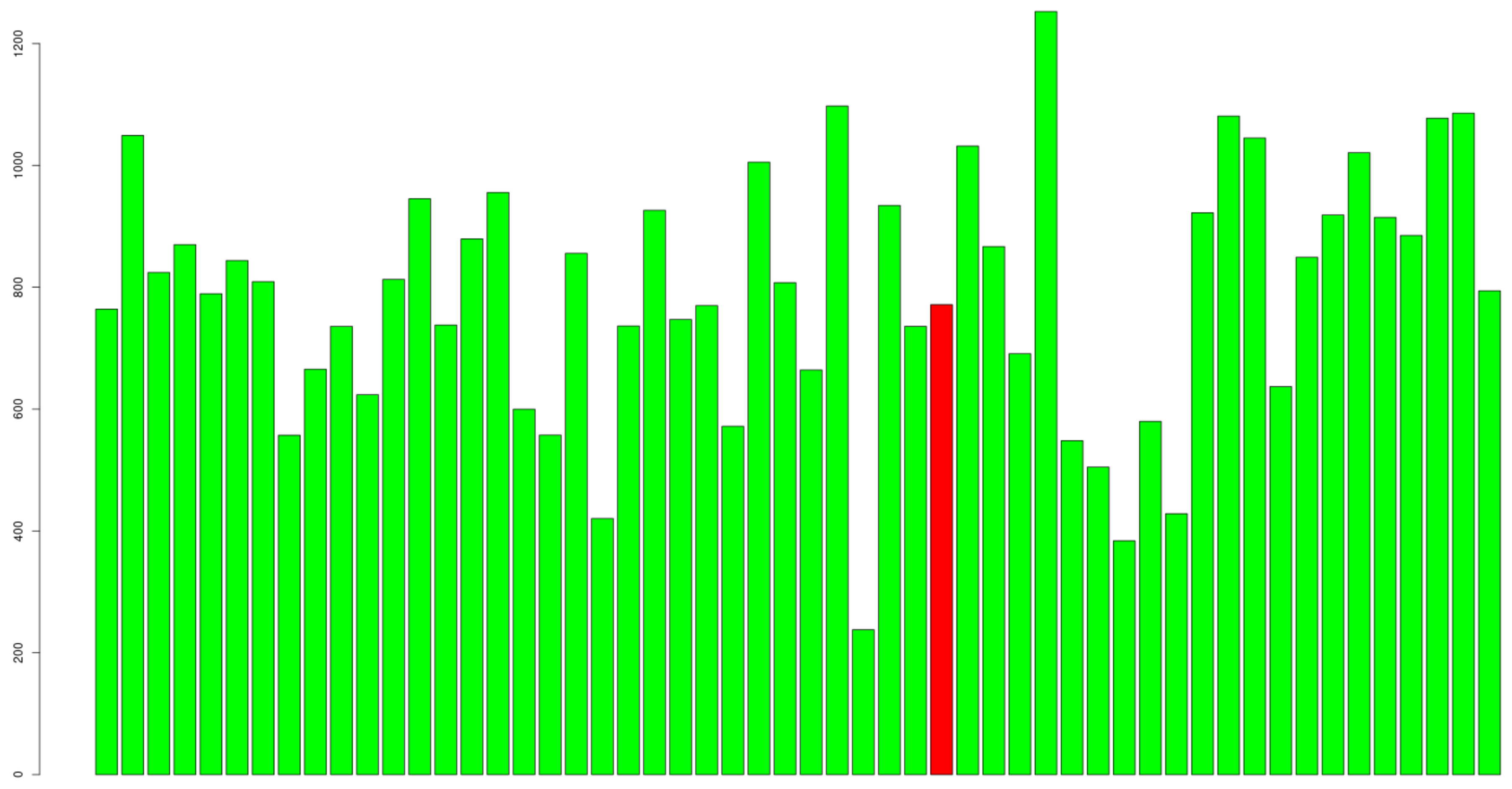

Supplementary figure 2. Expression levels (FPKM) of *MYBPC3* in myocardium across the patient cohort. Red color denotes level of expression in a patient with a mutation with a predicted NMD consequence.

Supplementary table 1. Primer and probe sequences used for allelic discrimination real-time PCR assay and Sanger sequencing

Methods	Gene and CDS position	SNPs	Nucleotide Sequence 5′→3′	Size (bp)
TaqMan allelic discrimination assay	MYBPC3 c.3697C>T	rs397516037	Forward primer: GAAACACAGATGTGTCTCCCTGGG	262
			Reverse primer: CAGGCGGCACTCACACCG	
			Probe-Allele-C: FAM-AGAGTCAACACTCCCTGCTTGCTGAACATG-BHQ1	
			Probe-Allele-T: VIC-CAGAGTCAACACTCCCTACTTGCTGAACATGC-BHQ2	
Sanger sequencing	MYBPC3 c.1855G>T	NA	Forward primer: TGCAGGGTCCACAAACTGAC	205
			Reverse primer: GAAGAGCAAGTAGCACGGGG	
	MYH7 c.1357C>T	rs121913625	Forward primer: CTCAATATGGGTCTCTCCTCCACC	226
			Reverse primer: GAGCGAGTGAGTGATTGTTCTCC	
	MYH7 c.1987C>T	rs397516127	Forward primer: TCCTCTCTTCTTCCTGCATCTCTT	163
			Reverse primer: GCCTCACCTGGAGACTTTGT	
	MYH7 c.2572C>T	rs2754158	Forward primer: CTACTTCAAGATCAAGCCGCTGC	216
			Reverse primer: TGGAAGAGCCAACAGTAGCCC	
	MYH7 c.3116A>G	rs199573700	Forward primer: GAACAACAAAATCACCAAGTCAGGA	264
			Reverse primer: TTGTACTGTTATGGGCTGGGGA	
	MYH7 c.4133A>G	NA (ID: 2769580)	Forward primer: ATGACTGCGACCTGCTGC	249
			Reverse primer: CATCGCCTGTGTGGGATCTG	
	MYH7 c.5134C>T	rs121913650	Forward primer: AACGACGACCTGAAGGAGAACA	269
			Reverse primer: CAGAGCAGGAAAAGCATTGAGCA	
	MYH7 c.5333A>T	NA	Forward primer: CCGACCCGATCCAGACCAGTG	283
			Reverse primer: CATTCTCCAGCTCCCGCACC	

Supplementary table 3. Alignment rates for each alignment obtained

Patient_ID	Total_reads_count	Total_reads_mapped	Total_mapping_percent	Uniqely_mapped_read_count	Uniqely_mapped_read_percent	Multimapped read count	Multimapped_read_percent
2H	21284164	21167017	99.45	17182726	80.73	3984291	18.72
4H	26480813	26329604	99.43	20926322	79.02	5403282	20.4
5H	24548388	24396067	99.38	19536561	79.58	4859506	19.8
6H	26720094	26514245	99.23	21142839	79.13	5371406	20.1
7H	20320497	20179699	99.31	16082687	79.15	4097012	20.16
9H	22562913	22432690	99.42	18289447	81.06	4143243	18.37
10H	27901190	27664793	99.15	21111861	75.67	6552932	23.49
11H	30005066	29815976	99.37	22862988	76.2	6952988	23.17
12H	17794788	17682752	99.37	14371074	80.76	3311678	18.61
13H	25664343	25502845	99.37	19738436	76.91	5764409	22.46
15H	22232532	22087141	99.35	17706519	79.64	4380622	19.7
22H	22128723	21974747	99.3	17946149	81.1	4028598	18.2
24H	23026087	22853913	99.25	18347490	79.68	4506423	19.57
26H	25901101	25749048	99.41	20288544	78.33	5460504	21.08
27H	26546756	26396242	99.43	21567517	81.24	4828725	18.19
28H	23782927	23587363	99.18	15617103	65.67	7970260	33.52
29H	22824307	22596010	99	16899699	74.04	5696311	24.96
31H	21566261	21352567	99.01	16688392	77.38	4664175	21.63
32H	23877274	23632808	98.98	17711791	74.18	5921017	24.8
33H	23398748	23257185	99.39	18373119	78.52	4884066	20.87
34H	19866350	19744857	99.39	15903118	80.05	3841739	19.33
37H	21545719	21416442	99.4	16895084	78.42	4521358	20.98
38H	25381543	25231866	99.41	18261616	71.95	6970250	27.46
40H	15016634	14664464	97.65	12456457	82.95	2208007	14.7
45H	12365126	12125104	98.06	10520442	85.08	1604662	12.97
46H	13293012	13050715	98.18	11432327	86	1618388	12.17
47H	14802225	14554440	98.33	12152008	82.1	2402432	16.23
48H	14128727	13793950	97.63	12120324	85.78	1673626	11.84
51H	13278598	11935601	89.89	8830737	66.5	3104864	23.38
58H	13103226	12753655	97.33	10922289	83.36	1831366	13.98
59H	14709746	14495907	98.55	12266798	83.39	2229109	15.16
60H	13571154	13298120	97.99	11566488	85.23	1731632	12.76
61H	11909955	11583208	97.26	8977962	75.38	2605246	21.88
62H	11093651	10777872	97.15	8300440	74.82	2477432	22.33
63H	10827154	10464322	96.65	7203876	66.54	3260446	30.11
64H	10997166	10664234	96.97	8308793	75.55	2355441	21.42
70H	12091349	11775599	97.39	9329554	77.16	2446045	20.23
71H	10921002	10438909	95.59	7758407	71.04	2680502	24.55
72H	12299025	11983109	97.43	9356421	76.07	2626688	21.35
73H	10951541	10582348	96.63	7166119	65.43	3416229	31.19
74H	12056880	11541514	95.73	8818339	73.14	2723175	22.58
75H	12042817	11740045	97.49	9010582	74.82	2729463	22.66
76H	12411046	11885734	95.77	10273566	82.78	1612168	12.99
77H	10418912	10034652	96.31	8726069	83.75	1308583	12.56
78H	11164276	10856932	97.25	8853866	79.31	2003066	17.94
79H	11051053	10638221	96.26	9179747	83.07	1458474	13.19
80H	10774824	10372589	96.27	8899493	82.6	1473096	13.68
81H	12218275	11933335	97.67	9512008	77.85	2421327	19.81
